# Construction of a human epidermal growth factor receptor 2-related gene risk model for predicting breast cancer prognosis

**DOI:** 10.3892/ol.2025.15414

**Published:** 2025-12-01

**Authors:** Limin Huang, Chunhong Xu, Yining Song, Furong Sun, Xuemei Sun, Hanyi Yao, Mingchen Liu, Nan Luo

**Affiliations:** 1 Department of Oncology III, Weifang Hospital of Traditional Chinese Medicine, Weifang, Shandong 261041, P.R. China; 2 Department of Breast Thyroid Surgery, Weifang Hospital of Traditional Chinese Medicine, Shandong 261041, P.R. China; 3 Intensive Care Unit, Weifang Hospital of Traditional Chinese Medicine, Shandong 261041, P.R. China; 4 First Clinical Medical College, Shandong University of Traditional Chinese Medicine, Jinan, Shandong 250013, P.R. China

**Keywords:** human epidermal growth factor receptor 2, breast cancer, prognosis, risk model, tumor microenvironment

## Abstract

The present study aimed to construct a human epidermal growth factor receptor 2 (HER2)-related gene risk model to predict breast cancer prognosis. Gene expression and clinical follow-up data were extracted from The Cancer Genome Atlas database, while the GSE7390 dataset was obtained from the Gene Expression Omnibus database. Prognostic and clinical feature analyses were performed. In addition, differentially expressed genes (DEGs) between HER2-negative and -positive groups were screened, followed by enrichment analysis. Subsequently, a prognostic model was established, and prognosis was predicted using a nomogram. In addition, the association of risk score with immunity was analyzed, and single-cell analysis was performed. Next, key genes were identified by reverse transcription-quantitive PCR (RT-qPCR) analysis. The results revealed that HER2 was significantly associated with estrogen receptor status, progesterone receptor status, N stage, American Joint Committee on Cancer stage, mutation count and tumor mutation burden of breast cancer. AS601245, AP.24534 and roscovitine were the top three chemotherapeutic agents showing the highest sensitivity differences between the risk groups. A total of 251 DEGs between HER2-negative and -positive groups were screened, which were found to be significantly involved in the Kyoto Encyclopedia of Genes and Genomes pathway of estrogen signaling, PI3K-AKT signaling pathway and chemical carcinogenesis-receptor activation. Eight prognostic gene models were constructed, and it was found that patients in the high-risk group had significantly shorter survival times than those in the low-risk group. A nomogram, incorporating risk groups and clinicopathological features, demonstrated strong predictive ability and high accuracy. The RT-qPCR results indicated that the expression of electron transfer flavoprotein subunit α, rap guanine nucleotide exchange factor-like 1, keratin 7, cluster of differentiation 24, proline rich 15-like, arachidonate 15-lipoxygenase type B, ELOVL fatty acid elongase 2 and C-X-C motif chemokine ligand 9 was consistent with the results of bioinformatic analysis. In conclusion, the HER2-related risk model and nomogram developed in the present study demonstrated high accuracy in predicting patient survival.

## Introduction

Breast cancer, a common type of cancer, is widespread among women (1). Globally, breast cancer accounts for approximately one-third of all malignant tumors in women, with its mortality rate representing ~15% of the total diagnosed cases (2). Breast cancer has surpassed lung cancer in terms of incidence and has become the leading form of cancer globally, far exceeding other types of cancer in women; the incidence rate of breast cancer is approximately 2-fold the combined incidence rate of cervical, ovarian and endometrial cancer (2). In China, ~429,105 new cancer cases and 43,780 mortalities due to breast cancer were reported in 2022, with an earlier onset age than that of Western countries, and the peak incidence was between 45 and 55 years of age (3). Although early detection and increased awareness of cancer prevention have led to decreased mortality, the disease's incidence rates rose by 1% each year from 2012 to 2021 (1). Thus, understanding the nosogenesis of breast cancer and identifying powerful new biomarkers are necessary for the treatment and prognosis of breast cancer.

Human epidermal growth factor receptor 2 (HER2) belongs to the same family of epidermal growth factor receptors (4). HER2 activation has been demonstrated to significantly promote the occurrence of cancer (5). HER2 is linked to unfavorable outcomes in human breast cancer, including increased risk of early recurrence and metastasis (6). HER2-positive breast cancer is a highly invasive malignant disease (7). Due to considering HER2 as a therapeutic target for breast cancer and advances in HER2-targeted therapy, the prognosis of patients with HER2-positive breast cancer has been changed, and with improved anti-HER2-targeted therapy, patient survival time has significantly increased (8,9). However, an increasing recognition of the frequent occurrence of tumors with low or heterogeneous HER2 exists (10). Thus, understanding HER2 is of utmost importance for the treatment of breast cancer.

The present study aimed to screen HER2-related indicators, build a prognostic risk model to predict breast cancer prognosis, and identify potential chemotherapeutics for HER2-positive breast cancer. The current findings provide a foundation for the treatment and prognosis of HER2-positive breast cancer.

## Materials and methods

### Data collection and preprocessing

The Cancer Genome Atlas (TCGA) database (http://gdc.cancer.gov) was used to retrieve TCGA-breast invasive carcinoma (BRCA) gene expression and clinical data. After data pre-processing, 741 TCGA breast cancer samples were obtained for subsequent analysis. In addition, clinical information from the GSE7390 dataset was acquired from the Gene Expression Omnibus (GEO) database (https://www.ncbi.nlm.nih.gov/). After excluding specimens with an overall survival (OS) time of 0 days or missing survival data (due to data unavailability or inability to obtain), a total of 198 breast cancer samples were included in the analysis.

### Prognosis and clinical feature analyses

Kaplan-Meier survival curves were generated using the R package ‘survival’ (Version 3.2-13; http://bioconductor.org/packages/survivalr/) (11), with statistical comparisons performed by log-rank test. The Kaplan-Meier analysis was employed to evaluate the association between different HER2 sample groups (negative and positive) and OS. In addition, the association between HER2 and clinical data of breast cancer cases was evaluated by χ^2^ or Fisher's exact tests.

### Tumor microenvironment (TME)

The Cell-type Identification By Estimating Relative Subsets Of RNA Transcripts (CIBERSORT) (https://cibersort.stanford.edu/index.php) (12), Single-sample Gene Set Enrichment Analysis (ssGSEA) (http://www.bioconductor.org/packages/release/bioc/html/ssGSEA.html) (13) and xCELL (https://github.com/dviraran/xCell) (14) algorithms were adopted to assess the score of immune cells according to the mRNA expression matrix of breast cancer samples. The ‘ESTIMATE’ package (http://127.0.0.1:29606/library/estimate/html/estimateScore.html) (15) in R was employed to obtain stromal, immune and ESTIMATE fractions. The expression data of checkpoint genes, human leukocyte antigen family genes and chemokine genes were extracted from the breast cancer expression data, and the differences in expression levels between negative- and positive-HER2 sample groups were compared using the Wilcoxon rank-sum test.

### Mutation analysis

Based on breast cancer sample mutation information, the mutation status of each gene in the samples was counted, genes were sorted in descending order by the number of mutations and then the TOP 20 genes with the highest mutations were selected for mutation display. Furthermore, the mutation frequency of the top 20 genes was analyzed using the ‘maftools’ package (Version 2.8.0) (https://bioconductor.org/packages/release/bioc/html/maftools.html) (16). In addition, the tumor mutation burden (TMB) of all cancer samples was determined, and the discrepancy in TMB values between the HER2-negative and -positive groups was analyzed.

### Drug sensitivity analysis

The sensitivity of all cases to chemotherapy agents was assessed based on data obtained from the Genomics of Drug Sensitivity in Cancer database (https://www.cancerrxgene.org/), and the half maximal inhibitory concentration (IC_50_) was quantified with the ‘pRRophetic’ package (https://github.com/paulgeeleher/pRRophetic) (17). Differences in the IC_50_ values of 138 chemotherapeutics between HER2-negative and -positive groups were analyzed using the Wilcoxon rank-sum test.

### Immunotherapy response

The Tumor Immune Dysfunction and Exclusion (TIDE) database (http://tide.dfci.harvard.edu/) was used to analyze the response of patients to immune checkpoint treatment, which was represented as the TIDE score. In addition, the immunophenoscore (IPS) was used to determine the scores of the four different immune phenotypes (namely, inhibitory cells, effector cells, antigen presentation and checkpoints). Furthermore, the Gene Set Variation Analysis algorithm was employed to evaluate the tertiary lymphoid structure (TLS) scores of TLS feature genes [such as CC motif chemokine ligand (CCL)2, CCL3 and CCL4]. Finally, the differences in TIDE, IPS and TLS scores between HER2-negative and -positive groups were analyzed using the Wilcoxon rank-sum test.

### GSEA

GSEA (http://bioconductor.org/packages/release/bioc/html/GSVA.html) (18) was used to assess the significant enrichment pathways of hallmark gene sets (h.all.v7.4. symbols) between HER2-negative and -positive groups with P*<*0.05 and |(Normalized Enrichment Score|>1.

### Identification of differentially expressed genes (DEGs)

To identify HER2-specific genes, differential expression analysis was performed between HER2-positive and HER2-negative samples using the ‘limma’ package (Version 3.34.7) (https:// bioconductor.org/packages/release/bioc/html/limma.html) (19). This analysis generated gene-specific P-values, logFC (log_2_ fold change), and other relevant metrics. Additionally, the limma package was employed to perform an empirical Bayes moderated t-test/F-test. The resulting P-values were sorted and adjusted using the Benjamini and Hochberg method to obtain false discovery rate (FDR)/q-values, thereby controlling the overall FDR. DEGs meeting |log2 fold-change|≥0.585 and adj. P<0.05 were used for subsequent analyses. In addition, enrichment analysis of DEGs was performed through the ‘clusterProfiler’ package (Version 4.0.5) (http://bioconductor.org/packages/release/bioc/html/clusterProfiler.html) (20) in R, with a threshold of count >2 and adj. P<0.05.

### Construction of a prognostic risk model

Univariate Cox regression analysis of DEGs was performed using the Kaplan-Meier ‘survival’ package (http://bioconductor.org/packages/survivalr/) (11) with a critical value of P<0.05 to identify genes associated with prognosis. The least absolute shrinkage and selection operator (LASSO) is a dimensionality-reduction method that has shown advantages over regression analysis in evaluating high-dimensional data. LASSO is an improvement of linear regression that achieves feature selection and complexity control by adding a penalty term. For parameter optimization, LASSO provides two criteria: i) ‘min’ selects the model achieving minimum cross-validation error within allowable variance; and ii) ‘1se’ (one standard error rule) chooses the most parsimonious model whose performance is statistically indistinguishable from the ‘min’ model, effectively balancing accuracy and simplicity. This algorithm can determine the optimal penalty coefficient based on the minimum likelihood deviation and 10-fold cross validation (21). Therefore, LASSO (21) was used to identify key genes. The risk score was constructed using stepwise Cox regression analysis with the survminer package in R (Version 0.4.9) (https://cran.rstudio.com/web/packages/survminer/index.html). The formula is as follows: RiskScore=h (t,X)=h_0_ (t) × exp (β_1_X_1_ + β_2_X_2_ + ... + β_n_X_n_), where β indicates the regression coefficient; h_0_(t), the benchmark risk function; and h (t,X) the risk role related to X (covariate) at time t. All specimens were classified into high- and low-risk groups according to the median RiskScores. Survival analysis was performed using the Kaplan-Meier curve method with the log-rank test for statistical comparison between groups. To evaluate the diagnostic performance of feature genes, receiver operating characteristic (ROC) curves and the area under the curve (AUC) were plotted across all datasets, including both training and validation sets.

### Nomogram construction

The association between RiskScores and clinicopathological data (such as age and stage) was investigated, and univariate and multivariate Cox regression analyses were performed to screen for useful prognostic features, with a threshold of P<0.05. Next, the ‘rms’ package (Version 6.2-0) (https://cran.r-project.org/web/packages/rms/index.html) (22) was further utilized to construct a nomogram.

### Association analysis of RiskScore and immunity

The ‘ssGSEA’ and ‘CIBERSORT’ algorithms were employed to evaluate the proportional distribution of immune cells between different risk groups. Spearman correlation analysis was performed using the ‘ggcor’ package (Version 0.9.8.1) (https://pan.baidu.com/s/1S6w93IjfO6sU8IHGvvCa1w) (23) to assess the association between RiskScore and immune cell infiltration.

### Characteristics of key genes

The associations between the expression levels of key genes were determined with the ‘ggplot2’ package (Version 3.3.5) (https://github.com/tidyverse/ggplot2) (24), while the ‘RCircos’ package (Version 1.2.2) (https://github.com/hzhanghenry/RCircos) (25) was employed to draw the mutation gene plot of key genes. The Kaplan-Meier curve was used to assess the survival of patients. The log-rank test was employed to determine the statistical significance of differences between survival curves with a P-value threshold of <0.05.

### Single-cell analysis

Based on a single-cell RNA sequencing database focused on the TME accessed through the Tumor Immune Single-cell Hub (TISCH) database (http://tisch.comp-genomics.org), scRNA-seq data from GSE161529 (26) were analyzed. Detailed cell type annotation was performed at the single-cell level for subsequent analysis of specific gene expression across distinct cell populations (27).

### Reverse transcription-quantitative PCR (RT-qPCR)

Six HER2-negative and six HER2-positive breast cancer samples were collected from May 1, 2023, to December 31, 2023, at Weifang Hospital of Traditional Chinese Medicine (Weifang, China). The enrolled patients ranged in age from 32 to 69 years, and all samples were obtained through surgical resection. The Ethics Committee of Weifang Hospital of Traditional Chinese Medicine approved the present study (approval no. WF2023-428). All the participants provided written informed consent.

For RT-qPCR, total RNA was extracted from tissue samples using an RNeasy Kit (Qiagen, Inc. cat. no. 74104) according to the manufacturer's protocol. According to the manufacturer's instructions, the RNA samples were reverse transcribed into cDNA using an QuantiTect Reverse Transcription Kit (cat. no. 205311; Qiagen GmbH), and qPCR was performed using a CFX96 Bio-Rad system (Bio-Rad Laboratories, Inc.) with SYBR Green I fluorophore (cat. no. 1708882; Bio-Rad Laboratories, Inc.). The thermocycling conditions were as follows: Initial denaturation at 95°C for 3 min, followed by 40 cycles of 95°C for 10 sec and 60°C for 30 sec, with a final melting curve analysis from 65°C to 95°C in 0.5°C increments. The primer sequences, which were designed and synthesized by Sangon Biotech Co., Ltd., are listed in Table I. The internal reference gene for normalization was GAPDH. Each sample was analyzed in three technical replicates, and the relative levels of key genes were analyzed by the 2^−ΔΔCq^ method (28).

### Cell culture and transfection

The HER2-positive breast cancer cell lines BT474 (cat. no. TCHu143) and SKBR-3 (cat. no. TCHu225) (29), and the HER2-negative breast cancer cell line MDA-MB-468 (cat. no. TCHu136) (30) were purchased from The Cell Bank of Type Culture Collection of The Chinese Academy of Sciences. All cells were cultured in RPMI 1640 medium (cat. no. 11875093; Gibco; Thermo Fisher Scientific, Inc.) containing 10% fetal bovine serum (FBS; cat. no. 10099141; Gibco; Thermo Fisher Scientific, Inc.) in an incubator with 5% CO_2_ at 37°C. Cells at logarithmic proliferation stage were transfected. The ELOVL2 overexpression plasmid (OE-ELOVL2) and its negative control (NC-ELOVL2) were constructed using the pCMV6-AC-GFP vector backbone (cat. no. YC-13849RJ) by Shanghai Yaji Biotechnology Co., Ltd., and then transfected into cells using Lipofectamine^™^ 2000 (cat. no. 11668019; Invitrogen; Thermo Fisher Scientific, Inc.) at a concentration of 2 µg per 1×10^6^ cells. After 6 h of transfection, the complete medium was replaced and the cells were further cultured for 48 h. The transfected cells were then collected for subsequent experiments.

### Cell Counting Kit (CCK-8) cytotoxicity assay

BT474 and SKBR-3 cells were inoculated into 96-well plates with 1×10^4^ cells per well in a ~100 µl suspension, and cultured in a 5% CO_2_ incubator at 37ºC in triplicate wells. After the cells reached 80–90% confluence (as observed by phase-contrast microscopy), CCK-8 reagent (cat. no. CK04; Dojindo Laboratories, Inc.) was added, and incubated at 37°C for 2 h. Next, the absorption value at 450 nm was measured using a microplate reader (ELx800; BioTek Instruments; Agilent Technologies, Inc.), and the cytotoxicity curves of the cells at different times were plotted. The experiment was repeated three times independently.

### Western blotting

Cells were lysed using RIPA buffer (cat. no. P0013B; Beyotime Institute of Biotechnology) supplemented with 1 mM PMSF to extract the total proteins from each group. SDS-PAGE was performed with 30 µg protein loaded per lane on 10% polyacrylamide gels after determining the protein concentration in each group using a BCA kit (cat. no. 23225; Thermo Fisher Scientific, Inc.). After electrophoresis, the proteins were transferred onto a PVDF membrane, which was then blocked with 5% bovine serum albumin (MilliporeSigma) for 1 h at 37°C. Next, primary antibodies against PI3K (p110 alpha) (1:1,000; cat. no. 4255; Cell Signaling Technology, Inc.), phosphorylated (p)-PI3K recognizing p85 (Tyr458)/p55 (Tyr199) (E3U1H) (1:1,000; cat. no. 17366; Cell Signaling Technology, Inc.), AKT (pan) (11E7) (1:1,000; cat. no. 4685; Cell Signaling Technology, Inc.), p-AKT (Ser473) (D9E) XP^®^ (1:1,000; cat. no. 4060; Cell Signaling Technology, Inc.) and GAPDH (used as a loading control for normalization) (1:10,000; cat. no. 2118; Cell Signaling Technology, Inc.) were added and incubated at 4°C overnight. After that, the membranes were washed thoroughly to remove unbound primary antibodies, followed by incubation with the corresponding secondary antibody [Goat Anti-Rabbit IgG(H+L) (peroxidase/HRP conjugated); 1:10,000, cat. no. E-AB-1003; Wuhan Elabscience Biotechnology Co., Ltd.; and Goat Anti-Mouse IgG (H+L) (peroxidase/HRP conjugated); 1:10,000; cat. no. E-AB-1001; Wuhan Elabscience Biotechnology Co., Ltd.] at room temperature for 1 h. Next, a laser imager (Typhoon FLA 9500; GE Healthcare) was used for scanning with ECL Plus Western Blotting Substrate (Thermo Fisher Scientific, Inc.), and the gray values were analyzed after normalization to GAPDH as a loading control.

### Statistical analysis

R software (SPSS 22.0) and GraphPad software (version 7.0; GraphPad; Dotmatics) were used for data processing, statistical analyses and plotting. Survival curves were constructed using the Kaplan-Meier method, and the log-rank test was employed to compare survival differences between groups, provided the proportional hazards assumption held. For time-to-event data with intermediate events, the two-stage survival analysis method from the Two Stage Hazard Rate Comparison package (Version 0.1-6) was applied when the proportional hazards assumption was violated. One-way analysis of variance was performed to assess overall differences, followed by pairwise comparisons between groups using Tukey's honestly significant difference test. In Table II, continuous variables with normal distribution are presented as the mean ± standard deviation, with intergroup comparisons assessed using unpaired Student's t-test; while skewed data are reported as median (interquartile range) and were compared via Mann-Whitney U test or Kruskal-Wallis H test followed by Dunn's multiple comparison post hoc test. Categorical variables are expressed as counts and percentages, with comparisons analyzed using χ^2^ or Fisher's exact tests. Unpaired All statistical tests were two-sided and P<0.05 was considered to indicate a statistically significant difference.

## Results

### Prognosis and analysis of clinical features

**Figure 1.**
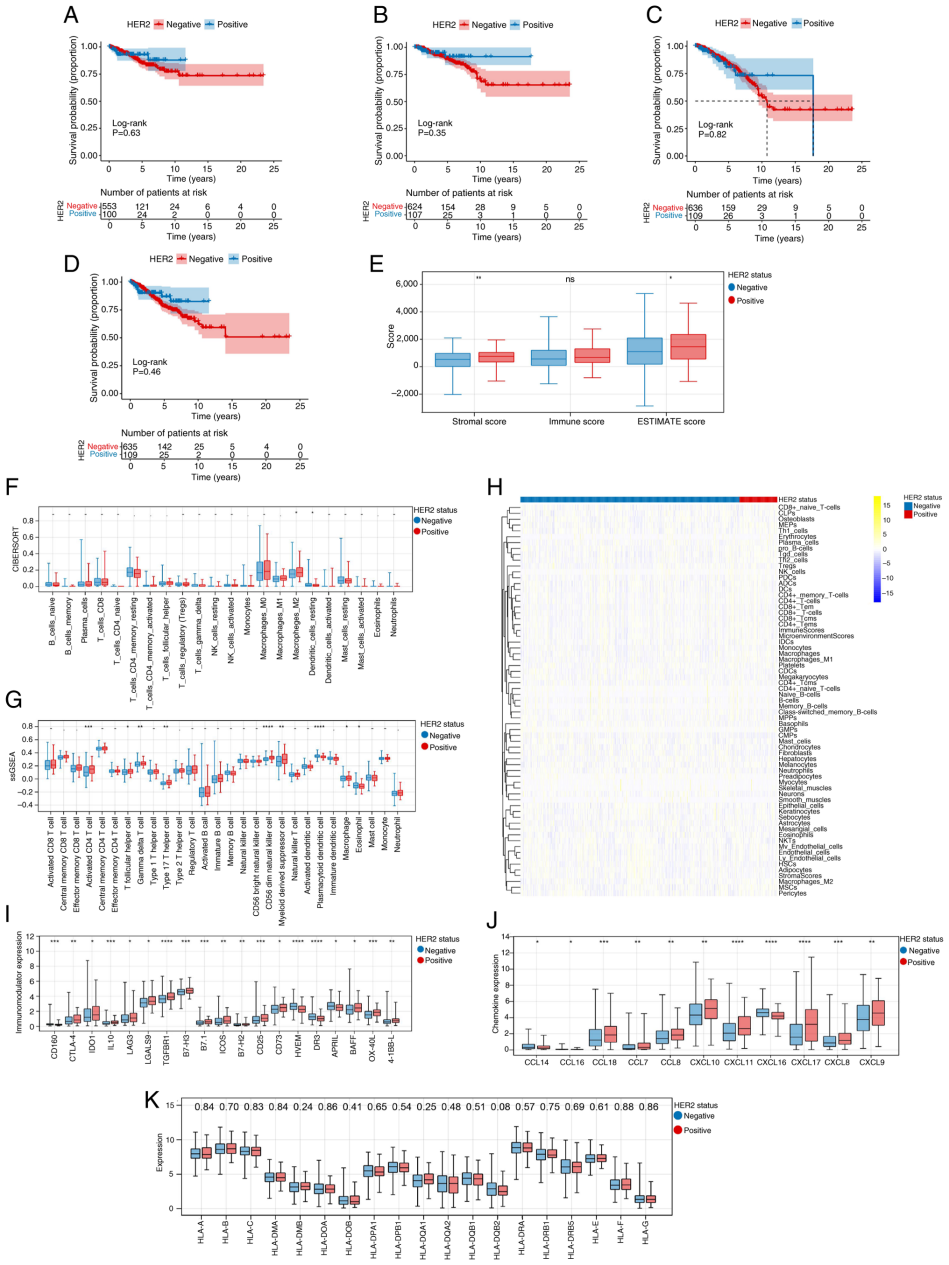
Prognosis, clinical features and tumor microenvironment analysis. Kaplan-Meier curves of (A) disease-free survival, (B) disease-specific survival, (C) overall survival and (D) progression-free survival in HER2-negative and positive groups. Note: Survival curves were constructed using the Kaplan-Meier method, and the log-rank test was employed to compare survival differences between groups, provided the proportional hazards assumption held. The two-stage survival analysis method from the two-stage Hazard Rate Comparison package was applied when this assumption was violated. (E) Differences in stromal, immune and ESTIMATE scores between HER2-negative and positive groups. The (F) ‘CIBERSORT’, (G) ‘Single-sample Gene Set Enrichment Analysis (ssGSEA)’ and (H) ‘xCELL’ algorithms revealed the fraction of immune cells in the HER2-negative and -positive groups. The numbers displayed in the yellow/blue legend represent the standardized expression levels (Z-scores). Differences in (I) Immunomodulator expression levels, (J) chemokine genes and (K) HLA family genes between HER2-negative and -positive groups. The numerical values displayed at the top of the panel refer to P-values. *P<0.05, **P<0.01, ***P<0.001, ****P<0.0001. ns, -, and . indicate no significant difference; HER2, human epidermal growth factor receptor 2; CIBERSORT, Cell-type Identification By Estimating Relative Subsets Of RNA Transcripts; ssGSEA, single-sample Gene Set Enrichment Analysis; NK, natural killer; T helper; HLA, human leukocyte antigen; CCL, Chemokine (C-C motif) ligand; CXCL, Chemokine (C-X-C motif) ligand; CLP, common lymphoid progenitors; MEP, megakaryocyte-erythroid progenitors; Th, T helper cells; Tgd, Tgd-like cells; iDC, immature dendritic cells; cDC, conventional dendritic cells; MPP, multipotent progenitors; GMP, granulocyte-macrophage progenitors; CMP, common myeloid progenitors; NKT, natural killer T-cells; mv, mv-like cells; HSC, hematopoietic stem cells; MSC, mesenchymal stem cells; Tregs, regulatory T cells.

As shown in Fig. 1A-D, no statistically significant differences were observed in prognosis between HER2-negative and -positive groups. Analysis of the association between clinical traits and HER2 suggested that HER2 was significantly associated with ER status, PR status, N stage, American Joint Committee on Cancer (AJCC) stage, mutation count and TMB (Table II).

### TME

Fig. 1E shows that the Stromal and ESTIMATE scores in HER2-positive group were higher than those in the HER2-negative group. Moreover, the ‘CIBERSORT’ algorithm revealed that the fraction of M2 macrophages and resting dendritic cells showed significant differences between the different HER2 groups (Fig. 1F). Additionally, the ‘ssGSEA’ and ‘xCELL’ algorithms revealed that there were 9 types (Activated CD4 T cell, T follicular helper cell, Gamma delta T cell, Type 17 T helper cell, CD56 bright natural killer cell, Myeloid derived suppressor cell, Plasmacytoid dendritic cell, Macrophage and Eosinophil) and 20 types of immune cells, respectively, which showed marked differences between different HER2 groups (Fig. 1G and H, respectively). The present study also identified 19 immune regulatory genes (Fig. 1I) and 11 chemokine genes (Fig. 1J) that exhibited remarkable differences between the HER2-negative and -positive groups. Fig. 1K demonstrates the differential expression of HLA family genes across distinct HER2 status groups.

### Mutation analysis, immunotherapy response and drug sensitivity analysis

**Figure 2.**
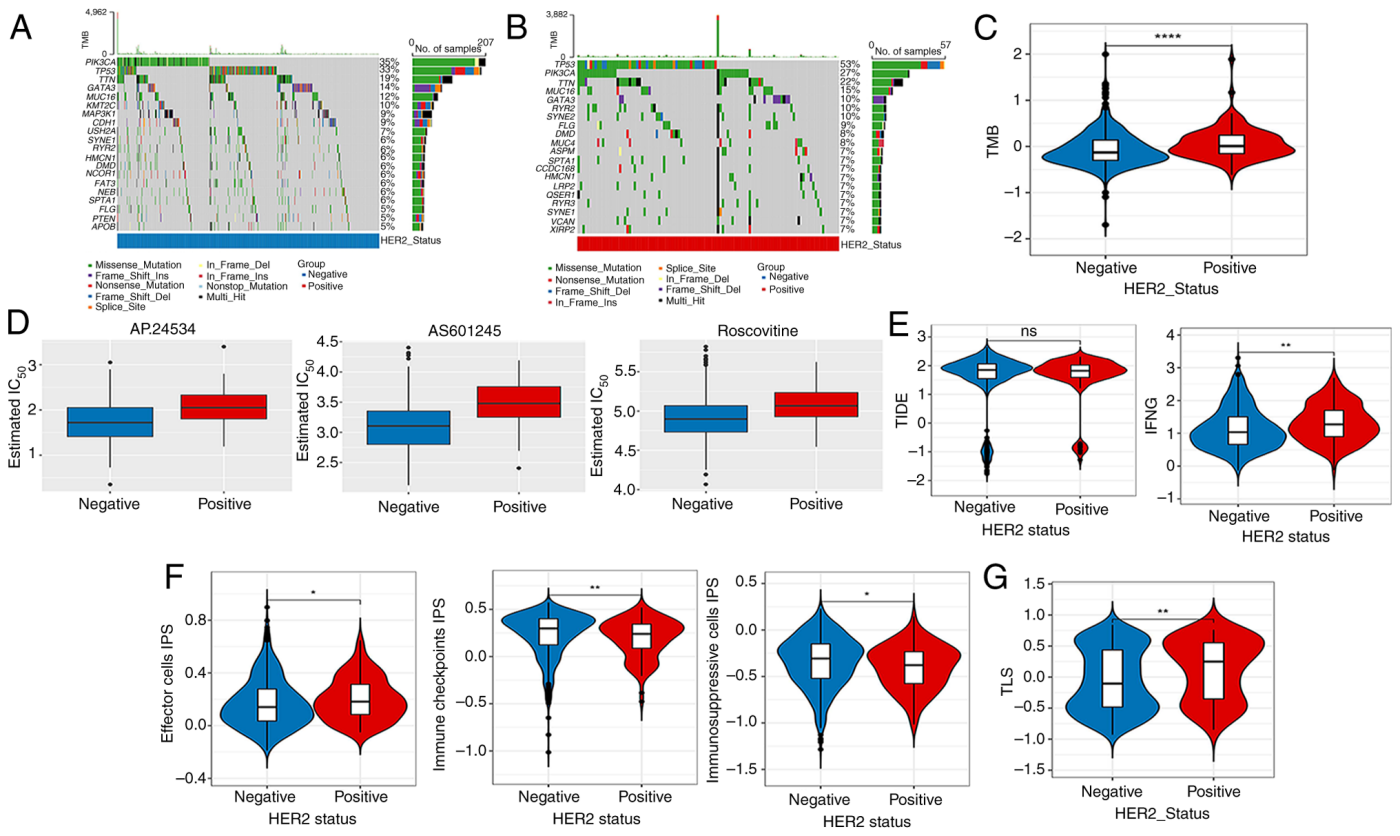
Mutation analysis, drug sensitivity analysis and immunotherapy response. Top 20 mutation genes in (A) HER2-negative and (B) -positive samples. (C) Differences in TMB values between the HER2-negative and -positive groups. (D) Top 3 chemotherapeutics with notable differences in their IC_50_ between the HER2-negative and -positive groups. Differences in (E) TIDE, IFNG, (F) IPS, (G) TLS scores between HER2-negative and -positive groups. ns, no obvious difference, *P<0.05, **P<0.01, ****P<0.0001. ns, no significant difference; TMB, tumor mutation burden; HER2, human epidermal growth factor receptor 2; Ins, insertion; Del, deletion; IC_50_, half maximal inhibitory concentration; TIDE, Tumor Immune Dysfunction and Exclusion; IFNG, interferon γ; IPS, immunophenoscore; TLS, tertiary lymphoid structure.

Based on the TCGA-derived mutational profiles, gene-specific mutation frequencies across breast cancer cohorts were systematically analyzed. Fig. 2A and B illustrates the hierarchical distribution of the top 20 most frequently mutated genes, with Fig. 2A and B demonstrating distinct mutational landscapes between HER2-negative and HER2-positive subtypes, respectively. Mutation analysis showed that, compared with those of the HER2-negative group, the TMB values of the top 20 genes in the HER2-positive group were higher (Fig. 2C). Fig. 2D shows the top three chemotherapeutic drugs with significant differences between the two groups. In addition, no significant difference was observed in the TIDE score among the HER2-positive and -negative groups, whereas the interferon γ score in the HER2-positive group was higher than that in the HER2-negative group (Fig. 2E). The data showed remarkable discrepancies in the effector cell, immunosuppressive cells and immune checkpoint IPS scores among the different HER2 groups (Fig. 2F). In addition, the TLS scores showed remarkable differences among the HER2-positive and -negative groups (Fig. 2G).

### Enrichment analysis

**Figure 3.**
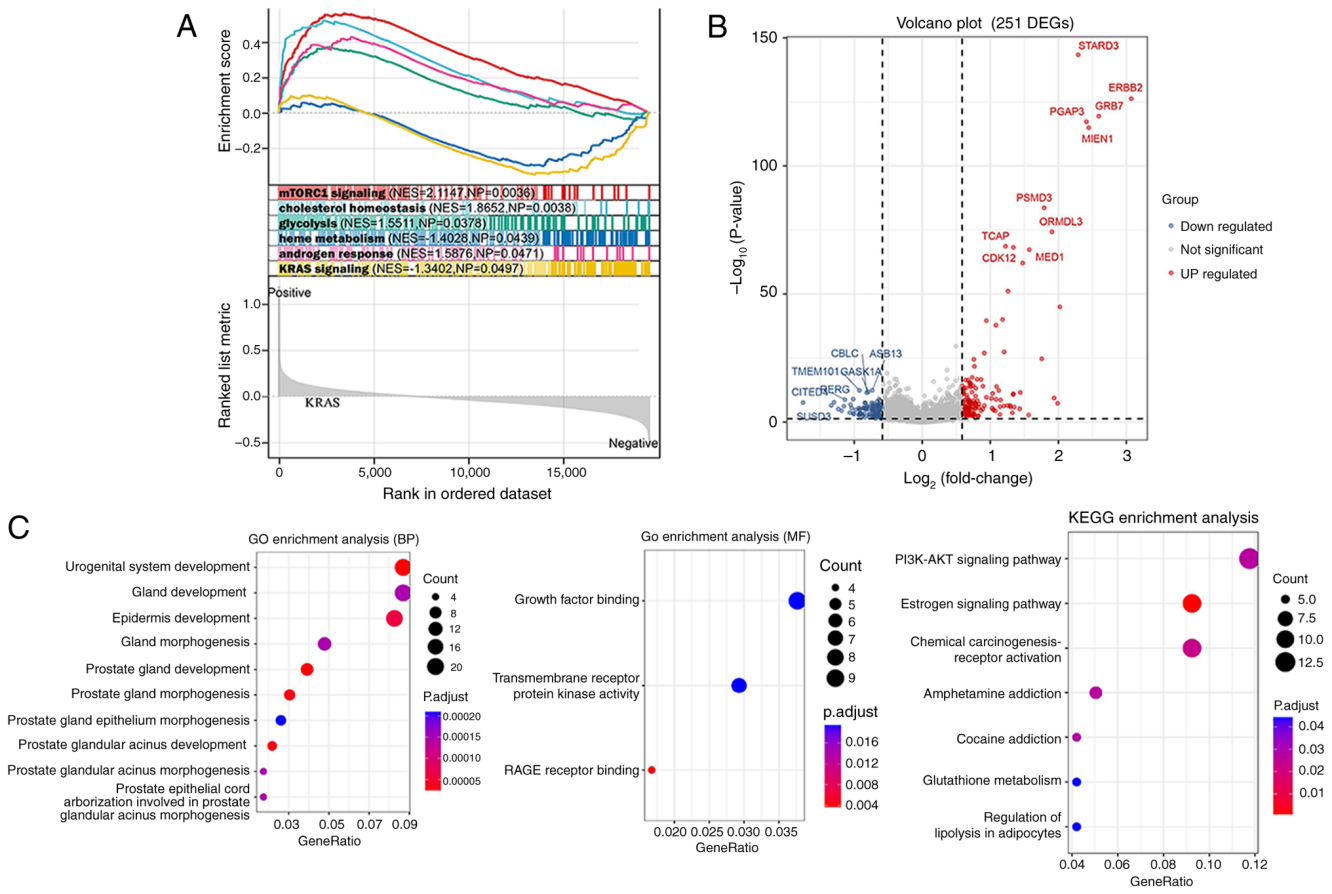
Identification of DEGs and enrichment analysis. (A) Gene Set Enrichment Analysis. (B) Volcano plot of DEGs between human epidermal growth factor receptor 2-negative and -positive groups. (C) Functional enrichment of DEGs. DEGs, differentially expressed genes; NES, normalized enrichment score; NP, normalized P-value; GO, Gene Ontology; BP, biological process; MF, molecular function; p.adjust, adjusted P-value; KEGG, Kyoto Encyclopedia of Genes and Genomes.

GSEA identified six pathways of hallmark gene sets between different HER2 groups, namely, mTORC1 signaling, cholesterol homeostasis, glycolysis, heme metabolism, androgen response and KRAS signaling (Fig. 3A). A total of 251 DEGs between different HER2 groups were screened (Fig. 3B), and these DEGs were enriched in the GO terms of urogenital system development and RAGE receptor binding, and were involved in the following KEGG pathways: Estrogen signaling pathway, chemical carcinogenesis-receptor activation and PI3K-AKT signaling pathway (Fig. 3C).

### Construction and validation of a prognostic risk model

**Figure 4.**
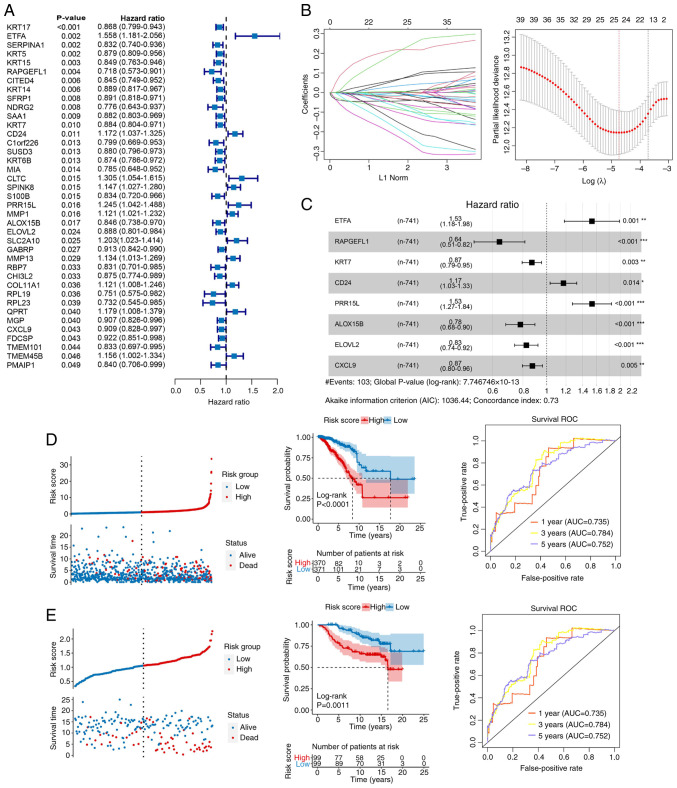
Prognostic model established in the current study. (A) Prognosis-related genes identified. (B) Characteristic genes screened by least absolute shrinkage and selection operator. (C) Stepwise Cox regression analysis. (D and E) RiskScore distribution, survival status, prognosis and ROC curves in (D) The Cancer Genome Atlas and (E) the GSE7390 dataset. *P<0.05, **P<0.01, ***P<0.001. AUC, area under the curve; ROC, receiver operating characteristic.

A total of 39 prognostic related genes were screened from the 251 DEGs (Fig. 4A). Subsequently, LASSO regression analysis was performed using univariate Cox regression with a P-value threshold of <0.05, which narrowed down the candidates to 25 genes (Fig. 4B). Moreover, eight key genes were identified after stepwise Cox regression analysis (Fig. 4C): ETFA, RAPGEFL1, KRT7, CD24, PRR15L, ALOX15B, ELOVL2 and CXCL9. A risk model was constructed based on the aforementioned key genes. As shown in Fig. 4D and E, compared with the low-risk group, patients in the high-risk group had a notably poorer prognosis (P<0.01), and AUC of the ROC curve was 0.735, 0.784 and 0.752 for the 1-, 3- and 5-year survival, respectively, in TCGA dataset (Fig. 4D), vs. 0.787, 0.796 and 0.771, respectively, in the GSE7390 dataset (Fig. 4E).

### Nomogram

**Figure 5.**
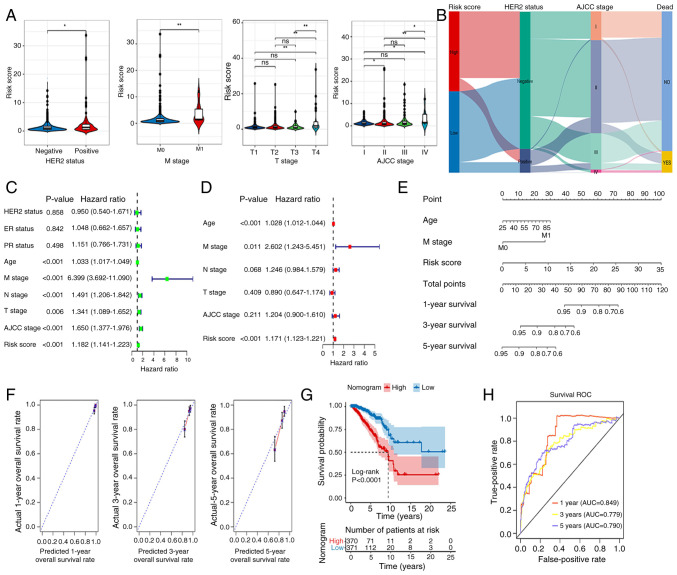
Construction of a nomogram. (A) Differences in RiskScore between different HER2-status groups and clinical features. (B) Association between HER2 and RiskScores. (C) Univariate and (D) multivariate analyses of clinical data and risk group. (E) Nomogram for predicting 1-, 3- and 5-year overall survival. (F) Calibration curve analysis of nomogram. (G) Kaplan-Meier analysis of the nomogram. (H) AUCs for predicting 1-, 3- and 5-year OS. *P<0.05, **P<0.01. ns, no significant difference; HER2, human epidermal growth factor receptor 2; ER, estrogen receptor; PR, progesterone receptor; M, metastasis; N, node; T, tumor; AJCC, American Joint Committee on Cancer; OS, overall survival; AUC, area under the curve; ROC, receiver operating characteristic.

Fig. 5A shows marked differences in Risk score between different HER2 groups (positive vs. negative), M stage (M0 vs. M1), T stage (T1 vs. T4, T2 vs. T4, T3 vs. T4) and AJCC stage (I vs. II, I vs. IV, II vs. IV, III vs. IV). Fig. 5B demonstrates that the high-risk group exhibits a significant tendency toward HER2-negative cases, whereas the low-risk group shows a higher proportion of HER2-positive cases. The HER2 status indirectly influences survival outcomes by affecting AJCC staging. HER2-negative cases are more likely to be classified as early-stage (I/II), associated with higher survival rates, while HER2-positive tumors tend to progress to advanced stages (III/IV), linked to elevated mortality risks. AJCC stage is strongly associated with survival outcomes: stages I, II, and III primarily exhibit NO (survival), whereas stage IV predominantly presents YES (death). Univariate Cox regression analysis was performed on clinical factors and risk score of the samples. Factors with P<0.05 were selected for subsequent multivariate Cox regression analysis to identify significant independent prognostic factors. Age, risk score and M stage were considered as independent prognostic factors (Fig. 5C and D). These factors were then used to build a nomogram, demonstrating that each variable independently contributed to survival probability prediction. Increased age corresponded with lower survival rates, advanced M stage significantly reduced survival probability, and higher risk scores were associated with poorer prognoses. Total points provided an intuitive prediction of outcomes, such as the 5-year survival rate. Model validation revealed high discriminatory ability (C-index=0.748) (Fig. 5E). The survival rate model predicted by the nomogram shows overall consistency with the actual survival rates for 1-, 3- and 5-year predictions. Specifically, the 1-year survival rate predictions are closely clustered around the ideal dashed line, indicating better consistency. The 3-year survival rate data points are more dispersed but still remain around the dashed line, demonstrating good consistency. However, the 5-year survival rate data points exhibit increased dispersion, with some deviating from the dashed line, leading to a decline in consistency. Overall, the consistency decreases as the predicted time span increases (Fig. 5F). Fig. 5G shows the marked association of these factors with patient outcomes. The ROC for the nomogram suggested that the AUCs at 1, 3 and 5 years were 0.849, 0.779 and 0.790, respectively (Fig. 5H).

### Association analysis of immunity and RiskScore

**Figure 6.**
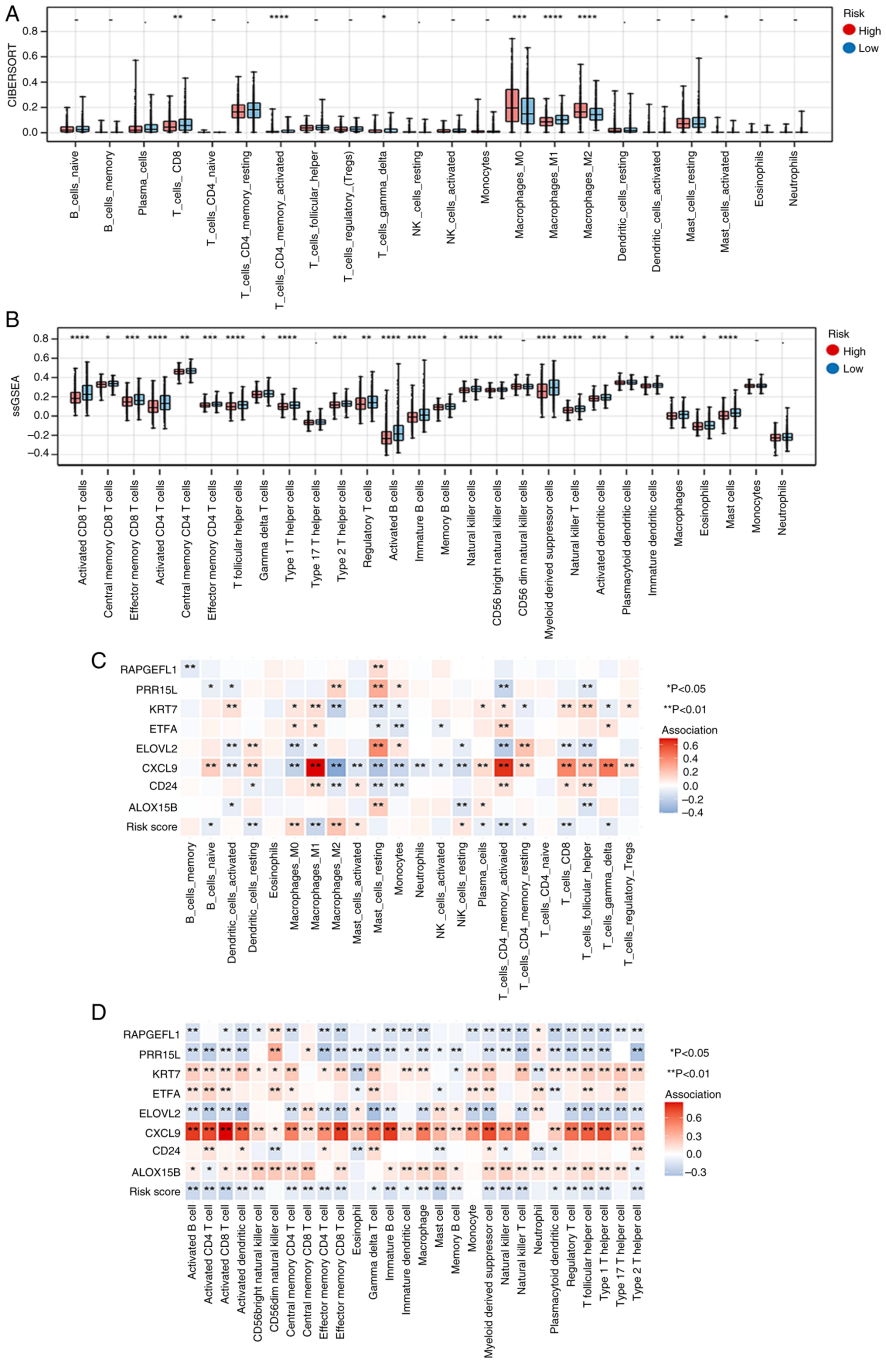
Association analysis of immunity and RiskScore. The (A) ‘CIBERSORT’ and (B) ‘ssGSEA’ algorithms were adopted to compare the score differences of 22 and 28 immune cells between the high- and low-risk groups. Association between 22 immune cell types (C), 28 immune cell types (D) and eight key genes. *P<0.05, **P<0.01, ***P<0.001, ****P<0.0001. -, no significant difference; ssGSEA, single-sample Gene Set Enrichment Analysis; Tregs, regulatory T cells; NK, natural killer.

A significant difference was observed in the proportions of 7 and 24 immune cell types between the high- and low-risk groups according to the results of ‘CIBERSORT’ and ‘ssGSEA’ analyses, respectively (Fig. 6A and B, respectively). The associations of 22 and 28 immune cell types with eight key genes are shown in Fig. 6C and D, respectively.

### Characteristics of crucial genes

**Figure 7.**
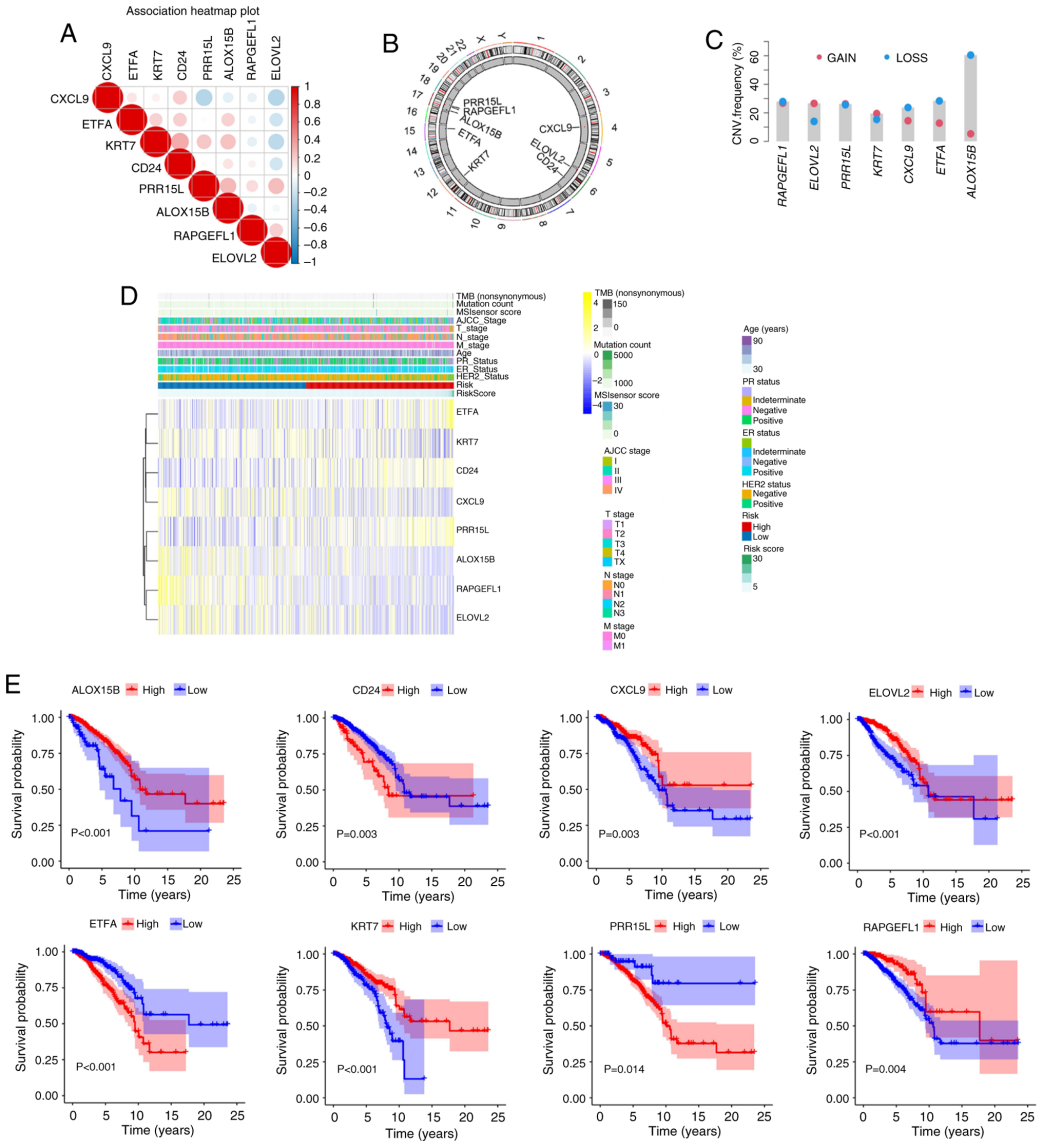
Characteristics of key genes (ETFA, RAPGEFL1, KRT7, CD24, PRR15L, ALOX15B, ELOVL2 and CXCL9). (A) Analysis of the association between the expression levels of the eight key genes and the prognosis of patients with breast cancer was analyzed revealed a positive trend in general. (B) CNV changes of the above eight key genes on chromosomes. (C) CNV changes (gain and loss trends) of the aforementioned eight key genes (there is no relevant information about gene CD24 so a graph cannot be drawn). (D) Heatmap of the expression of eight crucial genes. (E) Survival analysis patients with different levels of expression of the aforementioned eight crucial genes. Note: Survival curves were constructed using the Kaplan-Meier method, and the log-rank test was employed to compare survival differences between groups, provided the proportional hazards assumption held (ETFA, RAPGEFL1, KRT7, PRR15L, ALOX15B, and CXCL9 genes). For time-to-event data with intermediate events, the two-stage survival analysis method from the Two Stage Hazard Rate Comparison (TSHRC) package (Version 0.1-6) was applied when the proportional hazards assumption was violated (CD24 and ELOVL2 genes). CNV, copy number variation; TMB, tumor mutation burden; M, metastasis; N, node; T, tumor; AJCC, American Joint Committee on Cancer; HER2, human epidermal growth factor receptor 2; ER, estrogen receptor; PR, progesterone receptor. ETFA, electron transfer flavoprotein subunitα; RAPGEFL1, rap guanine nucleotide exchange factor-like 1; KRT7, keratin 7; CD24, cluster of differentiation 24; PRR15L, proline rich 15-like; ALOX15B, arachidonate 15-lipoxygenase type B; ELOVL2, ELOVL fatty acid elongase 2; CXCL9, C-X-C motif chemokine ligand 9.

Spearman's correlation test was utilized to analyze the association between the expression levels of the aforementioned eight key genes and the prognosis of patients with breast cancer was analyzed revealed a positive trend in general (Fig. 7A). Based on the circular graph, the chromosomal localization distribution and copy number variation (CNV) status of 8 model genes were visually mapped, clearly presenting the positions of each gene on the chromosome and their CNV alteration characteristics (Fig. 7B). The gain and loss trends are displayed in Fig. 7C. Fig. 7D shows heatmaps of the aforementioned eight key genes. Survival analysis showed that the prognosis of patients with high expression of CD24, ETFA and PRR15L was worse than that of patients with low expression, whereas RAPGEFL1, KRT7, ALOX15B, ELOVL2 and CXCL9 showed the opposite trend (all P<0.05; Fig. 7E).

### Single cell analysis

**Figure 8.**
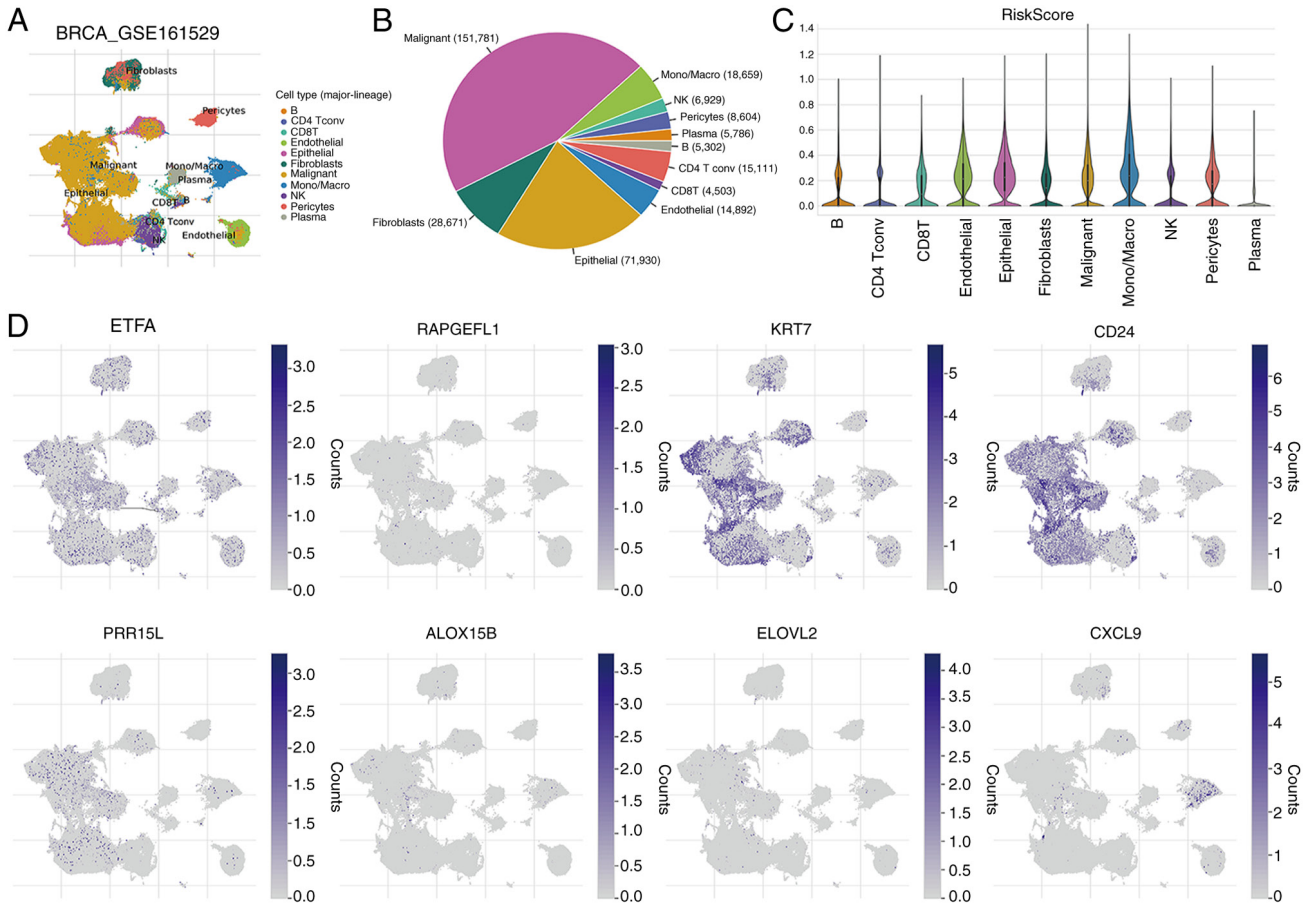
Single cell analysis. (A) Levels of eight key genes in 11 cell types based on the single-cell dataset GSE161529. (B) Distribution and percentage of 11 cell types. (C) Distribution of RiskScore in the above 11 cell types. (D) Expression pattern of the aforementioned eight key genes in immune cells. NK, natural killer; BRCA, breast invasive carcinoma; Tconv, Tconvoluted; Mono/Macro, monocyte/macrophage; ETFA, electron transfer flavoprotein subunitα; RAPGEFL1, rap guanine nucleotide exchange factor-like 1; KRT7, keratin 7; CD24, cluster of differentiation 24; PRR15L, proline rich 15-like; ALOX15B, arachidonate 15-lipoxygenase type B; ELOVL2, ELOVL fatty acid elongase 2; CXCL9, C-X-C motif chemokine ligand 9.

The GSE161529 single-cell dataset was used to explore the expression of the aforementioned eight crucial genes in the immune microenvironment, and the results revealed the involvement of 11 cell types (Fig. 8A). The distribution and percentage of these cell types are displayed in Fig. 8B. The largest proportions corresponded to malignant and epithelial cells. Fig. 8C shows the distribution of the RiskScore in the 11 cell types. In addition, immune microenvironment analysis revealed that the aforementioned eight key genes were expressed in various immune cells (Fig. 8D).

### Validation results

**Figure 9.**
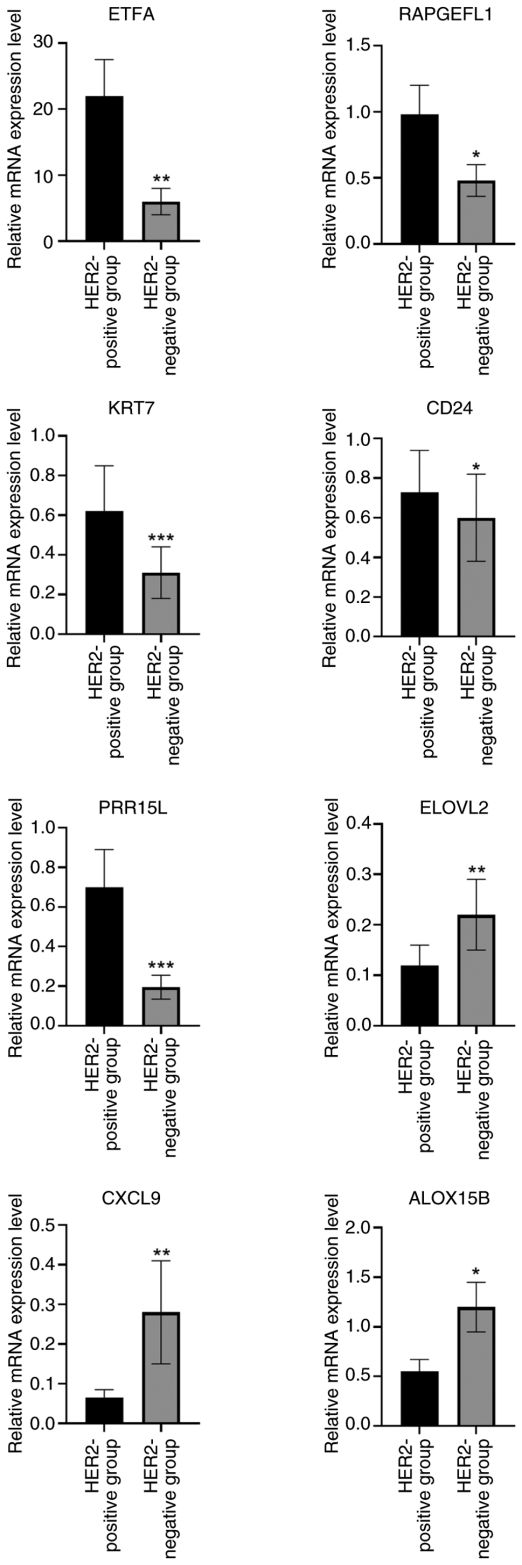
RT-qPCR was performed to validate the expression of eight crucial genes (ETFA, RAPGEFL1, KRT7, CD24, PRR15L, ALOX15B, ELOVL2 and CXCL9). *P<0.05, **P<0.01, ***P<0.001. HER2, human epidermal growth factor receptor 2; ETFA, electron transfer flavoprotein subunitα; RAPGEFL1, rap guanine nucleotide exchange factor-like 1; KRT7, keratin 7; CD24, cluster of differentiation 24; PRR15L, proline rich 15-like; ALOX15B, arachidonate 15-lipoxygenase type B; ELOVL2, ELOVL fatty acid elongase 2; CXCL9, C-X-C motif chemokine ligand 9.

RT-qPCR was performed to validate the expression of eight crucial genes (ETFA, RAPGEFL1, KRT7, CD24, PRR15L, ALOX15B, ELOVL2 and CXCL9). As shown in Fig. 9, the assays confirmed that ETFA, RAPGEFL1, KRT7, CD24 and PRR15L were significantly upregulated in the HER2-positive groups, whereas ELOVL2, CXCL9 and ALOX15B were significantly downregulated, compared with the HER2-negative group. ELOVL2 is a novel tumor suppressor with low expression in breast cancer (31). In prostate cancer, high expression of ELOVL2 suggests improved prognosis, and small hairpin RNA targeting ELOVL2 promotes cell proliferation, colony formation, migration and invasion, as well as the growth of subcutaneous xenografts by activating the PI3K/AKT signaling pathway (32). Therefore, ELOVL2 was selected in the present study for follow-up experiments.

### ELOVL2 overexpression inhibits the proliferation of HER2-positive breast cancer cells by inhibiting the PI3K-AKT pathway

**Figure 10.**
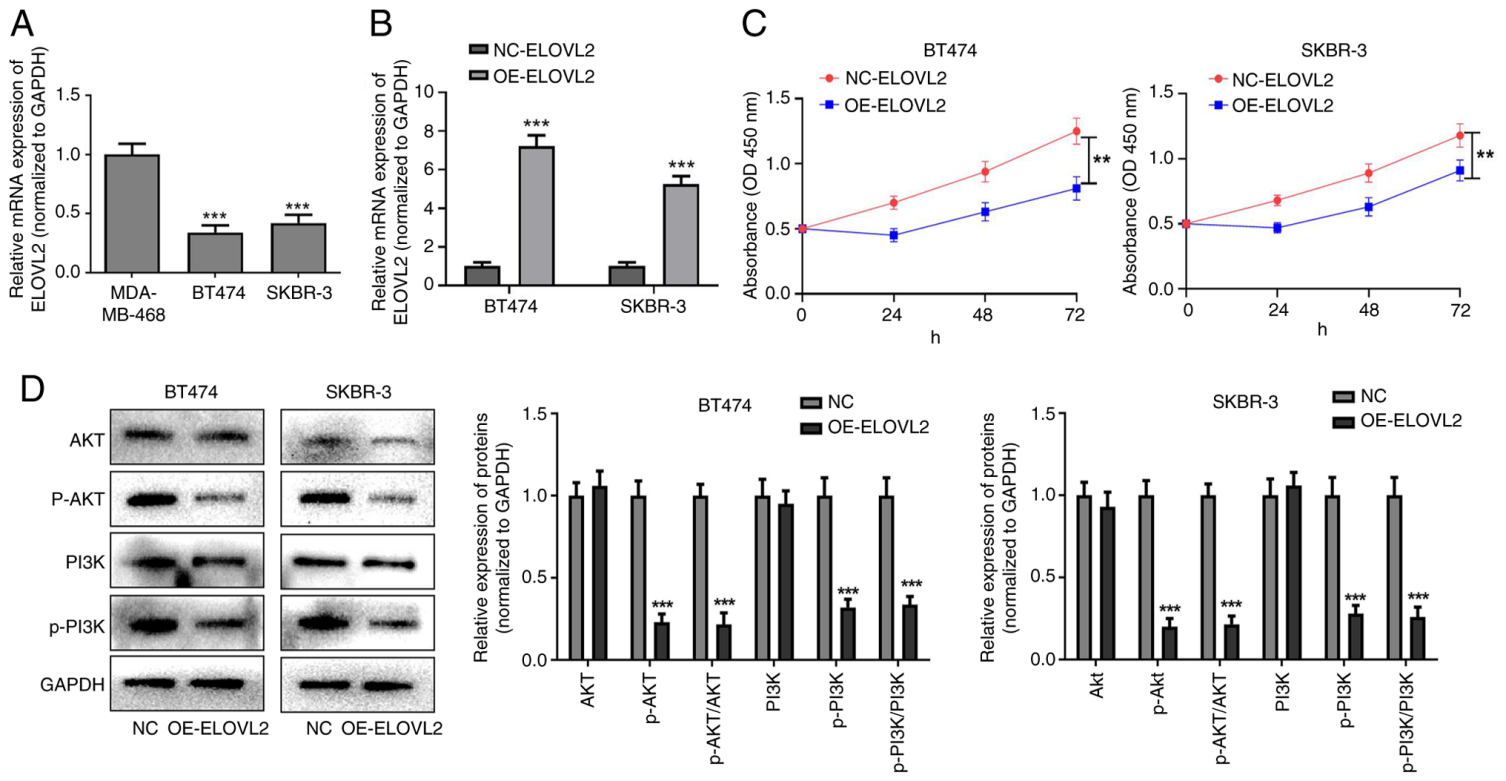
OE-ELOVL2 inhibits the proliferation of human epidermal growth factor receptor 2-positive breast cancer cells. (A) Expression level of ELOVL2 in MDA-MB-468, BT474 and SKBR-3 cells. (B) Reverse transcription-quantitative PCR was used to detect the transfection efficiency of OE-ELOVL2 in BT474 and SKBR-3 cells. (C) OE-ELOVL2 inhibited the proliferation of BT474 and SKBR-3 cells. (D) Effect of OE-ELOVL2 on the levels of PI3K, p-PI3K, AKT and p-AKT in BT474 and SKBR-3 cells. **P<0.01, ***P<0.001. OD, optical density; p, phosphorylated; NC, negative control; OE, overexpression.

The RT-qPCR results showed that the expression of ELOVL2 was significantly lower in BT474 and SKBR-3 cells than in MDA-MB-468 cells (P<0.001; Fig. 10A). In addition, RT-qPCR revealed that OE-ELOVL2 significantly increased the expression of ELOVL2 in BT474 and SKBR-3 cells, suggesting successful transfection (P<0.001; Fig. 10B). Furthermore, the CCK-8 proliferation assay showed that OE-ELOVL2 significantly inhibited the proliferation of BT474 and SKBR-3 cells (P<0.01; Fig. 10C). Western blot analysis showed no significant difference in the expression levels of PI3K or AKT in the OE-ELOVL2 group compared to the negative control group, whereas overexpression of ELOVL2 could decrease the levels of p-PI3K and p-AKT proteins. In addition, compared with the corresponding control group, the p-AKT/AKT and p-PI3K/PI3K ratios in the OE-ELOVL2 groups of both BT474 and SKBR-3 cell lines were significantly decreased (P<0.001; Fig. 10D). In conclusion, ELOVL2 overexpression inhibits HER2-positive breast cancer cell proliferation by inhibiting the PI3K-AKT pathway.

## Discussion

The oncogenic potential and activation of HER2 have been well established in various human tumors (33). In the present study, HER2 expression was significantly associated with ER status, PR status, N stage, AJCC stage, mutation count and TMB in breast cancer. Therefore, the molecular mechanisms underlying the involvement of HER2 in breast cancer were investigated. A total of 251 DEGs between HER2-negative and-positive groups were screened, and these DEGs were significantly enriched in the KEGG pathways of estrogen signaling pathway, chemical carcinogenesis-receptor activation and PI3K-AKT signaling pathway. Numerous studies have shown that these pathways are strongly associated with tumor progression. The PI3K/AKT signaling pathway is activated through the generation of 3′ p-phosphoinositides, which plays a crucial role in multidrug resistance in several cancer types such as breast cancer (34,35). The chemical carcinogenesis receptor activation pathway is involved in several diseases (36,37). Estrogen is involved in the metabolism of normal physiological processes and diseases, and the metabolic profile of endogenous breast cancer subtypes changes according to estrogen receptor expression (38). Therefore, it can be proposed that HER2-related DEGs participate in breast cancer progression through these pathways. However, this conclusion needs to be verified experimentally.

The TME plays a major role in tumorigenesis (39,40). Stromal and ESTIMATE scores, known as prognostic factors for tumors, are strongly linked to the tumor immune microenvironment, with higher scores associated with worse overall survival (41,42). The current results align with the above findings, showing that stromal and ESTIMATE scores in the HER2-positive group were increased compared to those in the HER-negative group. Furthermore, the immune cell scores of the HER2-negative and -positive groups were calculated. The ‘CIBERSORT’ and ‘xCELL’ algorithms revealed that the fraction of M2 macrophages showed marked differences between different HER2 groups. An increasing body of evidence indicates that M2 macrophages promote tumor growth and invasion (43–45). Yamaguchi *et al* (46) reported that M2 macrophages are involved in the development of peritoneal dissemination in gastric cancer. Additionally, the present study also identified 19 checkpoint genes, and 11 chemokine genes exhibited notable differences between the HER2-negative and -positive groups. Therefore, it can be considered that these immune cells, 19 checkpoint genes and 11 chemokine genes may participate in the development of HER2-positive breast cancer, providing reliable targets for immunotherapy of HER2-positive breast cancer.

The IC_50_ differences of 138 chemotherapeutic agents between HER2-negative and -positive groups were analyzed, and the top three chemotherapeutic agents with significant differences were identified, including AS601245, AP.24534 and roscovitine. Previous evidence suggests that activation of c-Jun N-terminal kinase (JNK) can promote tumor progression and is implicated in several types of tumors (47,48). AS601245 is an inhibitor of JNK signaling (47), and Luo *et al* (49) suggested that AS601245 may be a new inhibitor of breast cancer. AP.24534, known as ponatinib, is a pan-fibroblast growth factor receptor inhibitor (50). Ponatinib is currently used for the treatment of chronic myeloid leukemia (51), and is presently in clinical trials as an anticancer drug (52). Roscovitine is a small molecule that inhibits the activity of cyclin-dependent kinases by competitively binding to ATP-binding sites (53). Previous studies have confirmed that roscovitine blocks the cell cycle of cancer cells (54,55) and has synergistic effects with other anticancer drugs (56). In the present study, the IC_50_ values of the aforementioned top three chemotherapy drugs were higher in the HER2-positive group than in the HER2-negative group. Thus, AS601245, AP.24534 and roscovitine show promise for clinical application in HER2-positive breast cancer treatment.

In addition, a prognostic risk model was constructed using ETFA, RAPGEFL1, KRT7, CD24, PRR15L, ALOX15B, ELOVL2 and CXCL9. Numerous studies have demonstrated the importance of these genes in various cancer types. Chen *et al* (57) revealed that ETFA expression was upregulated in colorectal cancer, and that NBPF4 suppressed the progression of colorectal cancer by controlling the activity of EZH2-associated ETFA. Zhou *et al* (58) observed the amplification of *RAPGEFL1* in HER2-positive gastric cancer samples. KRT7 belongs to the family of genes known as keratins, which are highly expressed in numerous types of cancer and facilitate tumor progression (59,60). CD24 regulates the physiological activity of cancer cells (61). Furthermore, Li *et al* (62) reported that p53 induced iron death in bladder cancer cells by stimulating the lipoxygenase function of ALOX15B via suppression of SLC7A11. Hu *et al* (32) showed that ELOVL2 inhibited cell invasion, migration and proliferation in prostate cancer by modulating the activity of the cancer suppressor INPP4B. Seitz *et al* (63) demonstrated that CXCL9 inhibited tumor progression and enhanced the efficacy of anti-PD-L1 treatment in ovarian cancer. PRR15L is overexpressed in certain cancer types (64). More importantly, RT-qPCR analysis suggested that the expression of ETFA, RAPGEFL1, KRT7, CD24, PRR15L, ALOX15B, ELOVL2 and CXCL9 was consistent with the results of the bioinformatic analysis, indicating the reliability and robustness of the present study. Therefore, the above eight genes may be useful treatment targets for breast cancer.

Nomograms are valuable prognostic tools, including personalized applications and intuitive visual representations (65). In the current study, a nomogram was built using age, M stage and RiskScore, which demonstrated high accuracy in estimating the survival probabilities of individuals diagnosed with breast cancer, with ROC curves showing AUCs >0.75 at 1, 3 and 5 years, indicating an accurate predictive capability for survival. Wang *et al* (66) constructed a prognostic model for HER2-positive breast cancer based on nine autophagy-related genes (ARGs). The analytical methods used in that study are consistent with those used in the present study, and all the constructed prognostic models exhibited good accuracy and predictive ability. Zhao *et al* (67) constructed a prognostic model for predicting male patients with HER2-positive breast cancer, and the accuracy of this model was verified using calibration curves and decision curve analysis. The model genes found in the current study are different from those found in the models of the studies by Wang *et al* (66) and Zhao *et al* (67), which may be due to the different datasets selected. Wang *et al* (66) mainly studied ARGs, while Zhao *et al* (67) studied male patients with breast cancer, resulting in the selection of datasets and final model genes that are not consistent with the present study. Furthermore, a previous study found that a risk model composed of PTGDR, PNOC and CCL23 was helpful in predicting the prognosis of HER2-positive breast cancer, and that patients with low risk scores may benefit from immunotherapy (68). In general, the current and previous studies have shown that the constructed models are accurate and reliable, and can help clinicians select appropriate treatment strategies for HER2-positive breast cancer. However, these models require further investigations. Therefore, in the present study, the molecular mechanisms of the key gene ELOVL2 in HER2-positive breast cancer were preliminarily explored. The results showed that ELOVL2 overexpression inhibited the proliferation of HER2-positive breast cancer cells by inhibiting the PI3K-AKT pathway, suggesting that ELOVL2 is a potential target gene for the treatment of patients with HER2-positive breast cancer, laying the foundation for targeted therapy and improving the clinical adaptability of this model.

However, the current study has several limitations. First, the small sample size may have affected the accuracy of the results. Second, the molecular mechanisms of key genes affecting the prognosis of patients with HER2-positive breast cancer require further exploration. Third, the screened drugs must be validated experimentally. Therefore, future studies should collect additional cases, include a larger number of clinical samples, and conduct relevant clinical studies to provide effective personalized treatment plans or targeted therapies for patients and to improve their prognosis.

In conclusion, a valuable prognostic model that included eight HER2-related genes was developed in the current study. This model could accurately evaluate the survival rate of patients with HER2-positive breast cancer, and provide effective indicators or therapeutic targets for HER2-positive breast cancer. The present findings provide a new direction for the development of novel immunotherapeutic targets and personalized treatment for HER2-positive breast cancer.

## Figures and Tables

**Table I.** Primer sequences.

Gene	Sequence (5′-3′)
ETFA (F)	ACAAAGGCACTCTAGCCACC
ETFA (R)	GTCCAAGAGTGTCACCAGGG
RAPGEFL1 (F)	AGGGGCTGCTTCAAGAGGA
RAPGEFL1 (R)	CCCTGGTAAAGGGACTCGT
KRT7 (F)	CGGTGAGGACAAGGAACCTG
KRT7 (R)	CCTTGGAGCAGGAATCAGCA
CD24 (F)	TTCTCCAAGCACCCAGCA
CD24 (R)	TGGAATAAATCTGCGTGGGTA
PRR15L (F)	GACTTTAACACCCGCCTGGA
PRR15L (R)	TGAAGCGTCCTGAGTTGGAG
ALOX15B (F)	CCACCCTCTCTTCAAGTCCAC
ALOX15B (R)	CTTGGAGAAGATCTCTCTGACCC
ELOVL2 (F)	AAGCTGACATCCGGGTAG
ELOVL2 (R)	TGTCCACAAGGTATCCAGTT
CXCL9 (F)	GGCTTTGGAAGCCATGTGAT
CXCL9 (R)	GAAGAGCTGACTTGAATGAAGCAA
GAPDH (F)	GTCTCCTCTGACTTCAACAGCG
GAPDH (R)	ACCACCCTGTTGCTGTAGCCAA

F, forward; R, reverse. ETFA, electron transfer flavoprotein subunit α; RAPGEFL1, rap guanine nucleotide exchange factor-like 1; KRT7, keratin 7; CD24, cluster of differentiation 24; PRR15L, proline rich 15-like; ALOX15B, arachidonate 15-lipoxygenase type B; ELOVL2, ELOVL fatty acid elongase 2; CXCL9, C-X-C motif chemokine ligand 9; GAPDH, glyceraldehyde-3-phosphate dehydrogenase.

**Table II.** Association between clinical characteristics and HER2 expression.

Characteristics	Negative	Positive	Total	P-value
ER status, n (%)				6.00×10^−3^
Indeterminate	0 (0.00)	2 (0.28)	2 (0.28)	
Negative	138 (19.27)	31 (4.33)	169 (23.60)	
Positive	471 (65.78)	74 (10.34)	545 (76.12)	
PR status, n (%)				4.00×10^−3^
Indeterminate	4 (0.56)	0 (0.00)	4 (0.56)	
Negative	181 (25.31)	49 (6.85)	230 (32.17)	
Positive	423 (59.16)	58 (8.11)	481 (67.27)	
Age, years				0.71
Mean ± SD	57.83±13.15	58.37±13.21	57.91±13.15	
Median (range)	58.00 (26.00-90.00)	58.00 (27.00-90.00)	58.00 (26.00-90.00)	
M stage, n (%)				0.62
M0	620 (83.78)	107 (14.46)	727 (98.24)	
M1	12 (1.62)	1 (0.14)	13 (1.76)	
N stage, n (%)				3.00×10^−3^
N0	329 (44.16)	37 (4.97)	366 (49.13)	
N1	195 (26.17)	50 (6.71)	245 (32.89)	
N2	80 (10.74)	14 (1.88)	94 (12.62)	
N3	32 (4.30)	8 (1.07)	40 (5.37)	
T stage, n (%)				0.11
T1	180 (24.26)	19 (2.56)	199 (26.82)	
T2	368 (49.60)	74 (9.97)	442 (59.57)	
T3	63 (8.49)	10 (1.35)	73 (9.84)	
T4	22 (2.96)	6 (0.81)	28 (3.77)	
AJCC stage, n (%)				2.00×10^−3^
I	124 (17.06)	6 (0.82)	130 (17.88)	
II	348 (47.87)	71 (9.77)	419 (57.63)	
III	135 (18.57)	29 (3.99)	164 (22.56)	
IV	13 (1.79)	1 (0.14)	14 (1.93)	
DFS time, months				0.40
Mean ± SD	41.07±39.64	37.74±31.88	40.56±38.55	
Median (range)	28.50 (0.16-281.29)	23.34 (0.03-139.17)	28.44 (0.03-281.29)	
DFS status, n (%)				0.34
Disease-free	504 (77.18)	93 (14.24)	597 (91.42)	
Recurred/Progressed	49 (7.50)	7 (1.07)	56 (8.58)	
DSS tim, months				0.21
Mean ± SD	43.78±41.74	39.16±35.24	43.10±40.87	
Median (range)	31.36 (0.16-282.90)	24.79 (0.03-212.25)	31.00 (0.03-282.90)	
DSS status, n (%)				0.44
Alive	572 (78.25)	102 (13.95)	674 (92.20)	
Deceased	52 (7.11)	5 (0.68)	57 (7.80)	
MSIsensor score				0.75
Mean ± SD	0.65±2.01	0.44±0.61	0.62±1.87	
Median (range)	0.26 (0.00-32.92)	0.28 (0.00-4.09)	0.26 (0.00-32.92)	
Mutation count, n				3.00×10^−6^
Mean ± SD	81.15±271.60	116.03±413.31	86.26±296.53	
Median (range)	37.00 (2.00-5,400.00)	53.00 (17.00-4,259.00)	40.00 (2.00-5,400.00)	
OS time, months				0.21
Mean ± SD	43.77±41.71	39.16±35.24	43.10±40.84	
Median (range)	31.54 (0.16-282.90)	24.79 (0.03-212.25)	31.00 (0.03-282.90)	
OS status, n (%)				0.88
Alive	547 (73.42)	95 (12.75)	642 (86.17)	
Deceased	89 (11.95)	14 (1.88)	103 (13.83)	
PFS time, months				0.20
Mean ± SD	40.93±38.60	36.66±31.51	40.30±37.66	
Median (range)	28.77 (0.16-281.29)	21.83 (0.03-139.17)	28.44 (0.03-281.29)	
PFS status n (%)				0.45
Censored	546 (73.39)	98 (13.17)	644 (86.56)	
Progression	89 (11.96)	11 (1.48)	100 (13.44)	
TMB, n				3.10×10^−5^
Mean ± SD	2.75±9.02	3.94±13.76	2.92±9.86	
Median (range)	1.30 (0.00-180.83)	1.77 (0.00-142.67)	1.40 (0.00-180.83)	

Some sample-related indicators are missing (NA), so NA values were not included in the calculations. HER2, human epidermal growth factor receptor 2; ER, estrogen receptor; PR, progesterone receptor; SD, standard deviation; M, metastasis; N, node; T, tumor; AJCC, American Joint Committee on Cancer; DFS, disease-free survival; DSS, disease-specific survival; MSIsensor, microsatellite instability; OS, overall survival; PFS, progression-free survival; TMB, tumor mutation burden.

## Data Availability

The data generated in the present study may be requested from the corresponding author.
